# Exploring Partial Disclosure in Research: Challenges, Justifications, and Recommendations for Ethical Oversight

**DOI:** 10.1007/s41649-024-00311-7

**Published:** 2024-10-30

**Authors:** Ifeanyichukwu Akuma, Vina Vaswani

**Affiliations:** Center for Ethics, Yenepoya (Deemed to be University), Mangalore, Karnataka India

**Keywords:** Partial disclosure, Deception, Ethical concerns, Transparency, Research integrity

## Abstract

Deception in research is contentious, as ethical codes stress informed consent, yet complete disclosure may jeopardise validity. Indian Council for Medical Research (ICMR) guidelines classify deception into active, incomplete, and authorised forms. This study explores the ethical justification for incomplete (partial disclosure), permissible instances, and the dilemma faced by ethics committees in balancing scientific rigour and participant protection. The qualitative, non-experimental cross-sectional research, using in-depth interviews, identifies themes through thematic analysis. Findings reveal challenges for ethics committees, as incomplete information hampers understanding, amongst others. The paper proposes an ethics committee framework, urging researchers to minimise or avoid partial deception and recommending institutional awareness campaigns and standard operating procedures for minimal-harm studies using partial disclosure. Therefore, it proposes that partial disclosure should be justified by the 3Vs—value, validity, and veracity to preserve research integrity.

## Introduction

Partial disclosure refers to releasing or disclosing only certain pieces of information or details while withholding or keeping other information concealed. It implies that not all relevant or complete information is shared with the public or a specific audience. Partial disclosure can occur in research contexts. For example, researchers may disclose only specific information or findings while keeping other data or results concealed or unpublished. Such actions can be due to various reasons, including a lack of communication skills, ongoing research, patent applications, or the need for further analysis. It is important to note that partial disclosure can raise ethical concerns significantly when it hinders transparency, accountability, or the individual/public’s right to access information and may amount to deception.

Deception can be understood as any misleading claim or conduct that conceals reality through the sale of lies or withholding of essential information during data collection in research. Participants in such studies are unaware of the research’s objectives, design, technique, or procedure (Miller et al. 2008). A layperson’s perspective of deception varies with research expectations concerning what was told, what was done, and what was expected to have been done.

In the field of research, deception poses serious ethical concerns and can have significant negative consequences that violate ethical principles and standards, such as honesty, integrity, and transparency, undermining the trust and credibility of the scientific community and harming the well-being of participants and society (Kyi et al. 2023). Valuable resources can be wasted in deceptive research, including funding, time, and effort. Deception in research diverts resources away from genuine scientific inquiries and hampers the progress of knowledge and understanding in a particular field, leading to false or distorted conclusions. When researchers manipulate data or intentionally introduce bias, it becomes difficult to discern the actual facts or draw accurate conclusions based on the information (Brummett and Salter 2023). Deception can arise at various stages, such as study design, data collection, analysis, or interpretation. Both data manipulation and introducing bias can undermine the objectivity, validity, and reliability of research leading to deception if the intention is to mislead or misrepresent the research findings. They compromise the trustworthiness of scientific discoveries and can have serious consequences, including misinforming decision-making processes. Hence, even when researchers manipulate data or intentionally introduce bias, it is called deception. Misleading participants, leading them down the wrong path, and wasting their time and resources as they attempt to respond to flawed or fraudulent questions/research can hinder scientific progress and delay the development of effective solutions to real-world problems.

Nonetheless, there are pertinent guidelines on deception that provide a benchmark for the EC to make decisions. For example, the Council for International Organisations of Medical Sciences (CIOMS 2016) stated that active deception of participants is more controversial than withholding information. Researchers have a duty to avoid deception, influence, and coercion and reduce vulnerability because vulnerability increases the risk of harm and deception. Further asserted that social and behavioural scientists misinform participants intentionally to study the attitudes and behaviour of healthcare professionals in their natural settings (CIOMS 2016). Researchers must convince the research ethics committee that deception is necessary to obtain valid data and have significant social value. Researchers must obtain consent in advance for deception to maintain the scientific validity of research, and debriefing is essential to rectify the wrong of deception. Participants must be offered an opportunity to refuse to use their data, and in exceptional cases, the retention of non-identifiable information may be approved.

The Indian Good Clinical Practice (ICH 2023) states that ECs have a duty to ensure that there are no instances of unwarranted coercion, undue influence, or deception (Castelino et al. 2018). The participants should not be misled in any way. However, information may occasionally be suppressed until the study is over if it might compromise the reliability of the investon (P.24/49, ICH 2023). Also, the Indian Council of Medical Research is of the opinion that there should be a full committee review of research studies involving deception to ensure informed consent and for the larger public good. A two-step procedure for informed consent in research involving deception is necessary to avoid deception, influence, and intimidation. EC may approve withheld information. Deception can be used to violate human rights or consent. Furthermore, any deception in research should offer no more than minimal risk; have no negative effects on the welfare and safety of the subjects; be carried out only when the research cannot be done without lying; have a sufficient plan for debriefing the participants after the study is over, if necessary; communicate the research’s findings to the participants, if appropriate; and be properly reviewed by the EC (Behera et al. 2019).

More so, the American Psychological Association as amended (APA n.d.) guideline asserts that when employing deceptive methods, researchers have a duty to do so with caution and only when absolutely necessary (Kimmel 2012). Prospective participants should not be misled about study risks, and participants should be informed of the nature of deception after data collection. The tri-council policy statement (TCPS 2, 2022) and the APA Ethics Code adopt a more moderate stance, permitting deception when the advantages of the study outweigh the risks and the research question cannot be answered without the use of deception and full disclosure to participants upon study completion. However, it is pertinent to note that deception varies and is categorised differently by different scholars (Takeuchi et al. 2024); the concept differs from one discipline to the next, from one approach to the next, and from one field set to the next. In general, deception raises moral considerations about participants’ autonomy and respect for persons as restrictive problems with consent needs. Therefore, this study seeks to answer the question: what is the extent of ethical permissibility of using deception in research through partial disclosure? Hence, this article centres on exploring partial disclosure in research with the aim of uncovering its challenges, justifications, and recommendations for ethical oversight. By exploring the complexities inherent in navigating the ethical landscape of partial disclosure, the study shed light on the obstacles researchers encounter, the rationales they invoke, and proposed strategies for enhancing ethical conduct. Through an in-depth interview of EC members and thematic analysis, the research contributes to a deeper understanding of the ethical dimensions of partial disclosure and informs efforts to promote integrity, transparency, and accountability in research practise through a proposed standard operating procedure (SOP).

## Methodology

The study is non-experimental and descriptive cross-sectional research, adopting a qualitative approach to data collection. The researcher explored the experiences of the Ethics Committee members regarding deception through partial disclosure. Exploratory samples were used in the qualitative in-depth interviews. The chosen population for the study constituted all members of the Ethics Committee with variable interests and are registered with the Central Drug Standard Control Organisation (CDSCO). The Ethics Committee members shared similar characteristics, such as experience (not less than 1 year), and played specific roles in their respective professions. A one-on-one interview was conducted with eight participants. Data was collected and analysed independently and collectively. The analysed results were merged to compare, interrelate, and validate. The interpretation considered the equal emphasis on all interviews. The study was conducted only after approval from the Scientific Review Board and Ethics Committee.

## Result (Findings)

The perspectives of the participants in this study are denoted as P.1–P.8 (participant 1, 2, 3, 4, 5, 6, 7 & 8). Some participants’ views were presented in italics, indented, and enclosed in quotations in the discussion section.

Table 1 shows that eight participants partook in the interview. Six were female, and two were males accounting for three-quarters and one-quarter, respectively. It was felt that partial disclosure research is new and goes beyond hiding information regarding harmful impacts or other unexpected outcomes (P.1, 4 & 5). At the same time, few could not clearly differentiate between deception and partial or incomplete disclosure (P.6 & 7). Some considered the relationship between partial disclosure and deception as the level of information necessary to make an informed decision (P.2, 3 & 4). Furthermore, others relate partial disclosure to relevant disclosure, unwilling omission, willing commission, selective disclosure, purposeful action, and human error (P.4, 7 & 8).

**Table 1:** Demographic details for the in-depth interview

Ethics Committee members	Gender	Age	YOE	Roles
Participant 1	F	29	3	Layperson representative/language lecturer
Participant 2	F	46	5	Member secretary/basic scientist
Participant 3	F	46	3	Basic scientist/periodontist
Participant 4	F	42	3	Basic scientist/oncology
Participant 5	M	37	4	Scientific expert/homoeopathy
Participant 6	F	38	5	Bioethicist/physiology
Participant 7	M	47	6	Basic scientist/pharmacology
Participant 8	F	55	5	Clinician/paediatrics

### Coding Process

The analysis of the transcription generated six categories and 39 codes. The coding was done using the elective coding process. This process involves two cycles. In the first cycle, descriptive coding and in vivo coding were done to derive codes. Focused coding and thematic analysis were used in the second cycle to generate categories by splitting, splicing, and linking codes. The categories are described as follows;

#### Protocol-Related Issues

Deception could be intentional or unintentional. Intentional deception in research refers to situations where researchers deliberately mislead ECs or participants during a study design or implementation. While unintentional deception is a situation where researchers inadvertently mislead ECs or participants during a study design or implementation, despite not having any deliberate intention to deceive. Partial disclosure in research can occur due to various factors, including errors due to printing, technical languages (Jargon), flawed study design, inaccurate measurement tools, biassed interpretation of data, or incomplete understanding of the research topic. These, we collectively labelled as protocol-related issues.

Protocol-related issues refer to problems or challenges that ECs face in the context of reviewing protocol following a set guideline. These issues can occur during protocol design, where researchers do not fully disclose information (partial disclosure). Partial disclosure is only acceptable when it is necessary to protect privacy or maintain confidentiality (P.8) or serves the purpose of maintaining transparency and building trust with the involved parties (P.2). However, if partial disclosure has a detrimental effect on participant’s rights or well-being, it may not be acceptable (P.6).

Languages and terminologies used in science may vary significantly, and technical concepts may not have direct equivalents in some languages (P.1). Translators need to identify and disambiguate such technical terms to ensure the correct interpretation is conveyed in the target language. Using clear and simple words is key to understanding and averting the risk of miscommunication and inaccuracies in the protocol (P.4). Other issues identified included the lack of SOP for guiding the design and reviewing of deception research (P.7). Also, some protocol does not provide alternative options for the participant (P.5).

#### Deception-Related Issues

Deception-related issues in research refer to situations where researchers deliberately withhold or manipulate information in order to mislead participants or other researchers/ECs (P.2). Deception raises ethical concerns (P.6) and can undermine the participants’ ability to make an informed decision about participating in a study, as they are not fully aware of the true nature of the research (P.4). There should be a clear scientific rationale for using deception and a demonstration that the research objective cannot be achieved through alternative, non-deceptive methods (P.3). deception should never involve physical harm or pose a significant risk to participants (P.2). And researchers should provide participants with a complete explanation of the true purpose and procedures of the study (P.3). Also, if research is done to deceive, manipulate, or unfairly benefit one party at the expense of others, it becomes wrong (P.1 & 7).

#### Informed Consent–Related Issues

Issues related to informed consent arise when participants are not adequately informed or when coercion, manipulation, or undue influence is present (P.4). It is important for participants to have a clear understanding of the purpose, potential risks, benefits, and alternatives involved in any situation where participants’ consent is required (P.7). Deception should only be used when there are no feasible alternatives and when the potential benefits of the research outweigh the risks (P.4). Researchers should adhere to the ethical standards and guidelines established by their respective fields to minimise partial disclosure (P.3).

#### Motivational-Related Issues

Motivational-related Issues are pulling factors that necessitate deceiving or being deceived. The underlying intention plays a crucial role in determining the extent and nature of deception (P.3). However, when participants perceive potential benefits or rewards, they may be more inclined to engage in deceptive studies (P.1). Time constraints can influence the choice and effectiveness of deception. When researchers have limited time to plan and execute research, they may resort to simple or impulsive strategies (P.6). access to information, resources, or certain channels of communication can facilitate or impede deception (P.5 & 8). Lack of information, funds, clear vision, or goal makes it difficult to find the motivation to work towards completing a research project in an ethical manner. Also, weak policies or institutional loopholes may encourage deceptive practises, especially when the potential gains outweigh the perceived risks (P.6).

#### Researcher-Related Issues

This refers to practises attributed to the researcher leading to deception and may occur due to participant demand characteristics (social desirability bias) or methodological considerations. Some researchers are careless or negligent in their approach to research, and they may be more prone to cutting corners or using deceptive methods to achieve desired results (P.2). Researchers have a significant responsibility to conduct their studies with integrity and adhere to institutions or national ethical standards (P.1). This responsibility includes being transparent about the research process, obtaining informed consent from participants, accurately reporting findings, avoiding deceptive practises, and spelling-out risk to the participants (P.1 & 2). Nevertheless, engaging in deceitful practises carries risks such as damage to credibility, harm to participants, and potential legal consequences (P.1).

#### EC Role-Related Issues

EC role-related issues refer to challenges and concerns related to the role and responsibilities of the ethics committee functioning regarding research (deception studies). Ethics committees generally review protocols by considering the potential benefits of the study, the necessity, and the potential risks to participants (P.6). Deception can significantly impact the opinions and credibility of ECs if they are less vigilant in verifying the appropriateness of protocols (P.8). Hence, ECs should ensure researchers uphold moral and ethical principles in research to maintain the public’s trust (P.5). Ethical considerations are essential when utilising partial disclosure to avoid falsehood, infringements on privacy, or other violations of participants’ rights (P.7). More so, promoting ethical behaviour and decision-making within institutions or professional (research) contexts should not be undermined (P.3).

### Deception as a Part of Research Methodology

Deception as a part of research methodology is a controversial issue. In comparison, some participants (P.2, 3, 5, 7 & 8) argue that certain forms of deception can be justified ethically, for example, in certain circumstances where the deception involved is minimal and the potential scientific value of the study is significant. For example, suppose the research aims to understand subjective outcomes (like pain) and employs the placebo-control design (P.7), deception might be considered a technique to safeguard the research’s integrity and prevent participants’ responses from being influenced by knowledge of the study’s objectives (P.2, 3 & 8). The research benefits can exceed the deception if the researcher adequately debriefs participants after the research and minimises or eliminates potential harm or discomfort (P.5).

However, some participants (P.1 & 6) believe that any form of deception is ethically unacceptable and undermines the principles of autonomy and respect for individuals, violating their right to make informed decisions about participating in research (P.1). Deception can potentially lead to psychological distress, emotional discomfort, or loss of trust in participants. Even if the researcher conducts debriefing, it may not fully mitigate the adverse effects of the deception on individuals (P.6). The decision to use deception in research methodology requires careful consideration of the potential benefits, risks, and ethical implications.

Placebo and sham surgery are related to partial disclosure and deception in medical research and practise. When the researcher uses a placebo or sham surgery instead of actual treatment or surgical procedures, the participants may not always be wholly apprised of this fact by the researcher (P.8). However, the idea of placebo and sham surgery being related to partial disclosure and deception is subjective and varies in context.

Any substance or treatment that has no therapeutic effect on the patient’s condition is called a placebo. It is often used in clinical trials as a control group against which the effectiveness of a new treatment is measured. Placebos are typically given to participants who believe they are receiving active treatment, while another group receives the actual treatment being tested. Sham surgery refers to a type of control procedure used in clinical trials that mimics the real surgical intervention but does not involve the actual therapeutic component. The purpose of using placebos or sham surgeries is to assess the true efficacy of the treatment or surgical procedures to determine whether the observed benefits are attributable to the intervention or other factors, such as the placebo effect or natural healing processes. This could be considered acceptable deception (P.6 & 8).

## Discussion and Analysis

The discussion highlights the ethical rationale behind partial disclosure in research, the ethics committee’s stance on permitting partial disclosure in protocols, and the challenges encountered when protocols offer incomplete information. These themes were derived from the results to meet the research objectives. Thus, the discussion integrates perspectives from participants (P.1 to P.8) and correlates their views with related literature.

### Common Ethical Justification for Partial Disclosure in Research

#### Scientific Validity

Researchers using partial disclosure must be able to demonstrate that alternative methods, which do not involve deception, are not feasible or would compromise the validity of the study. Similarly, Cook and Toshio (2008) and Tai (2012) suggested that those who support deception must ensure the study’s credibility and relevance to the scientific community. In virtue ethics, the evaluation of an action depends on the character and intentions of the person performing it. Honesty and truthfulness are virtues to cultivate, as they contribute to trust, sincerity, and integrity (Kamtekar 2004; van Hooft 2014). On the other hand, deception goes against these virtues and undermines confidence in relationships. Virtue ethics does recognise that moral decision-making is complex and context-dependent (van Hooft 2014). Deception in certain situations may be considered justifiable or even virtuous, depending on the specific circumstances and intentions (Athanassoulis and Wilson 2009). For example, the validity of the research outcome provided the research does not expose participants to harm.

Scientifically valid research is crucial for producing reliable and credible results that can be used to inform knowledge and decision-making (P.6 & 8).

#### Informed Consent

Due to the nature of the research, the specifics of the partial disclosure may not be fully disclosed to participants until after their participation is complete, as this could compromise the integrity of the study. In the same vein, Tai (2012), Daniels et al. (2023), and Verbeke et al. (2023) emphasised the process of informed consent and the significance of important details. However, those studies were related to deception and consent but not to partial disclosure and ethics committee. In the principlism framework, deception undermines the principle of autonomy by limiting a person’s ability to make informed decisions about their health or participate in research voluntarily (Quante and Vieth 2002, O’Neill 2003, Wilson 2007). However, there are some situations where deception may be ethically justified, such as in certain research studies involving placebos or specific therapeutic interventions (Alfano 2015; Leder 2020). In these cases, the potential benefits to society or the individual may outweigh the harm caused by deception.

Researchers must provide informed consent for participants’ involvement in partial research, including any potential partial disclosure involved (P.1 & 4).

#### Essentiality

The essentiality of the research should be prioritise rather than research outcome or self-gain why adopting partial disclosure. Correspondingly, Strudler (2005), Oswald et al. (2014), and Leder (2020) identified bias control, unveiling sensitive topics, and educational purpose, amongst others, as essentials for adopting deception studies. According to Kant’s categorical imperative, people must only act on principles that can be applied universally without controversy. He made the case that deception regards people as tools rather than as ends in and of themselves (Fallis 2009). However, Kant acknowledged some unusual situations in which deception might be acceptable. He created the idea of the “right to lie”, which, in his opinion, may be used when deception is required to protect participants’ lives or avert grave harm. But he also cautioned that the one who lies (deceives) is responsible for all the consequences of the lie, even though unforeseeable (Korsgaard 1986; Wood 2011).

Before carrying out partial disclosure, researchers should evaluate its relevance, necessity, feasibility, and potential benefits compared to potential risks, whether the research question or objective addresses a significant gap in knowledge, has potential scientific or societal impact, or contributes to advancing a field of study (P.8).

#### Minimal Deception and Risk Assessment

This involves carefully balancing the potential benefits of the study against the potential harm (psychological, emotional, or social) caused by the partial disclosure (Kopelman 2004). Also, the use of partial disclosure in research should be subject to rigorous review by peers and ethical oversight committees (Baumrind 1985; Behera et al. 2019). The research design, methods, and ethical considerations should be evaluated to ensure that the use of partial disclosure is justified, necessary, and ethically sound. Similarly, Wendler (2020), in his work titled “The Permissibility of Deception in Riskier Research”, made the argument that when the study component about which participants are misled provides more than minimal risk, regulations should prohibit such study. In addition, the Ethics of Care generally promotes open and honest communication, as trust and empathy are considered crucial elements in maintaining caring relationships (Allmark 1995). Deception can be seen as a violation of these principles, as it undermines trust and can lead to harm or injustice (Huang et al. 2022). However, there may be situations where deception is justified within the Ethics of Care framework (Schermer 2007). For example, in cases where revealing the truth could cause unnecessary harm or distress to an individual, a researcher under the EC’s approval may temporarily withhold certain information (Meyers 2021). This is often called selective disclosure and is exercised to protect the individual’s well-being and reduce risk.

Partial disclosure should be kept to a minimum. Additionally, researchers should strive to design studies that use the least amount of partial disclosure necessary to achieve the research objectives (P.3, 6 & 7).

#### Debriefing and Full Disclosure

During the disclosure, participants should be informed about the true nature and purpose of the research, as well as the reasons behind the use of partial disclosure. This allows participants to make sense of their experiences and address any concerns they may have. In the same vein, Miller et al. (2008) stated that debriefing is a common ethical need for human research involving the use of deception as a means of fostering transparency. In the context of rights-based ethics, deception is generally considered ethically problematic. This is because deceiving others violates their right to truthful and honest communication or information. Rights-based ethics strongly emphasise the principle of honesty and integrity, considering it a fundamental moral duty to be sincere in our interactions with others (Raz 1984). However, rights-based ethics argue that there can be justifiable exceptions to the prohibition of deception. For example, deception may be morally permissible if it is necessary to prevent serious harm or protect fundamental rights (Brännmark 2017). This view is often associated with the “right to information”, which suggests that individuals have a right to be informed truthfully but not necessarily an absolute right to know everything.

Researchers have an obligation to fully disclose information to participants after their involvement in the study (P.2).

### Permissibility of Partial Disclosure for Protocols by the Ethics Committee

#### Reasonableness (Sensitive Information)

The ethics committee will assess the validity of the reasons given and make a decision based on the specific circumstances using a risk–benefit analysis. Alternatively, some research protocols may involve sensitive or confidential information that, if disclosed, could harm individuals or institutions involved (Strudler 2005; Oswald et al. 2014). In such cases, the ethics committee may consider partial disclosure to balance the need for transparency with the need to protect participants. Also, if the research protocol contains proprietary information or trade secrets, the ethics committee may allow partial disclosure to prevent the unauthorised use or dissemination of such information. In light of the above, Athanassoulis and Wilson (2009) argues that the relevant moral question that ethics committees ought to look into is not whether or not the participant from whom the knowledge is withheld is going to be deceived but rather look into the reasonableness of withholding knowledge from that participant who is considered to be deceived. Also, some proponents of cultural relativism argue that deceptive practises should be evaluated within the cultural context in which they occur. Cultural relativists say that what may be considered deceptive in one culture or context may not be viewed similarly in another (Seiter et al. 2002). Therefore, judgements about deception should be based on cultural norms and values rather than applying universal standards. However, there are limits to cultural relativism, such as actions that cause harm or violate fundamental ethical principles are often criticised regardless of cultural context.

The researchers must provide a clear and compelling justification for any request to withhold certain information from the protocol (P.7 & 8).

#### Transparency and Accountability (Trust and Scientific Integrity)

Researchers must be transparent about the use of partial disclosure in their research and provide a clear rationale for its necessity (Pascual-Leone et al. 2010). Alternatively, partial disclosure in research can erode trust amongst researchers, participants, and the wider public. Ethics committees need to consider the potential impact of such studies on public trust in research and ensure that the scientific integrity of the study is maintained. Brännmark (2017) argued in favour, stating that it is important for researchers to prioritise ethical principles like respect, honesty, and transparency when conducting medical and behavioural research of any sort to ensure the well-being and rights of participants are protected.

Ethics committees are responsible for holding researchers accountable and ensuring that research is conducted ethically and transparently (P.2 & 6).

#### Ethical Guidelines and Regulations

Most guidelines often provide specific guidance on deception but not on partial disclosure in research (Kimmel 2012; CIOMS 2016). Also, ethics committees should make sure that any proposed protocol aligns with these guidelines and does not violate ethical standards. ECs carefully evaluate the proposed procedures and assess whether the research meets the required ethical standards. Behera et al. (2019) compared the relevant details of the guideline for Medical Research in India (ICMR) from 2006 to 2017. Behera opined that guidelines do not only prescribe or offer specific information necessary for consent and research success but also play a vital role in identifying and minimising deceptive research practises.

Ethics committees follow established ethical guidelines and regulations when reviewing research proposals (P.3 & 7).

#### The Severity of the Risk and Potential Harm

Ethics committees must assess the severity of the risk and ensure that the potential risks of exposing participants to partial disclosure are reduced to minimal or mitigated (Wendler and Miller 2008). Also, Webster et al. (2018) argued that the contentious trade-off between the necessity of deception and its potential effects on participants is on the rise. It should be noted that deception research still carries risks, and researchers should be cautious of possible adverse outcomes. Utilitarianism generally prioritises the outcomes or consequences of an action over strict adherence to specific moral rules. Therefore, a utilitarian perspective may contend that such an action could be morally acceptable if deception in the research resulted in considerable benefits or value for numerous people (Pittenger 2002). However, this justification would depend on various factors, including the magnitude of the benefits, the likelihood of achieving those benefits, and the potential harms caused by the deception. It is important to note that utilitarianism does not endorse deception as a blanket principle. Instead, it considers the overall consequences and utility resulting from an action.

Partial disclosure in research has the potential to cause psychological, emotional, or other forms of harm to participants (P.6).

### The Difficulties the Ethics Committee Face When Dealing with Protocols That Only Partially Provide Information

#### Lack of Context and Ethical Implications

Without the complete context, it becomes challenging to make informed ethical deliberations, which may translate into giving incomplete information to participants altering their capacity to consent (Sears 2005). Researchers may provide incomplete information because he is afraid of losing research participants (Hammami et al. 2014). In the same vein, Athanassoulis and Wilson (2009) emphasised that the context of experimentation forms judgements concerning the circumstances within which withholding information from the participant is ethically acceptable in research. Incomplete information may obscure potential ethical concerns within the research protocol. Ethics committees rely on complete information to assess whether the proposed study complies with ethical principles such as autonomy, beneficence, non-maleficence, and justice. The absence of crucial details may hinder ECs’ ability to identify and address ethical issues adequately.

Incomplete information can make it difficult for the Ethics committee to fully understand the purpose, methods, potential risks and benefits of the research, and the validity and reliability of the data (P.1, 3, 7 & 8).

#### Institutional Arrangement

Institutions should have strong leadership that recognises the importance of ECs to reduce partial disclosure and sets a clear vision for research excellence and conduct (Mills et al. 2006). Furthermore, adequate time and remuneration are essential to support ECs’ activities. Therefore, institutions could actively seek external research grants and establish internal funding mechanisms to support research projects, ground-breaking discoveries, and advancements. Meeting the issue regarding the institutional arrangement and ECs functionality, Blunt et al. (1998) argued that for ECs to function better and effectively depends on the type of research community within which the committee operates.

In order to foster research success, institutions need to establish an effective institutional climate that supports, provides guidance and resources and promotes a culture of research excellence (P.1, 3, 5 & 6).

#### Lack of SOP for Partial Disclosure Research for EC

The lack of a standardised operating procedure (SOP) for partial disclosure research is indeed a challenge not only for ECs to typically review and provide oversight (Tiwari and Raman 2014) but also for researchers to compare and replicate studies (Dörries et al. 2011; Spicker 2022). Ultimately, this hinders the progress and reliability of research findings involving partial disclosure. However, it is important to note that the development of an SOP for partial disclosure research is essential, although a complex task. Deception itself is a multifaceted phenomenon with various forms and contexts (Takeuchi et al. 2024). As the field continues to evolve, collaborative efforts and discussions amongst Ethics Committee members and researchers would help to establish SOPs for the responsible and effective conduct of partial disclosure research. According to Thatte and Marathe (2017), he stated that standard operating procedures (SOPs) are a pressing issue in India and are essential for enhancing the performance of ECs and safeguarding research participants.

We do not have SOP for partial disclosure research, which makes it difficult in address it (P.1-8)

**Fig. 1:**
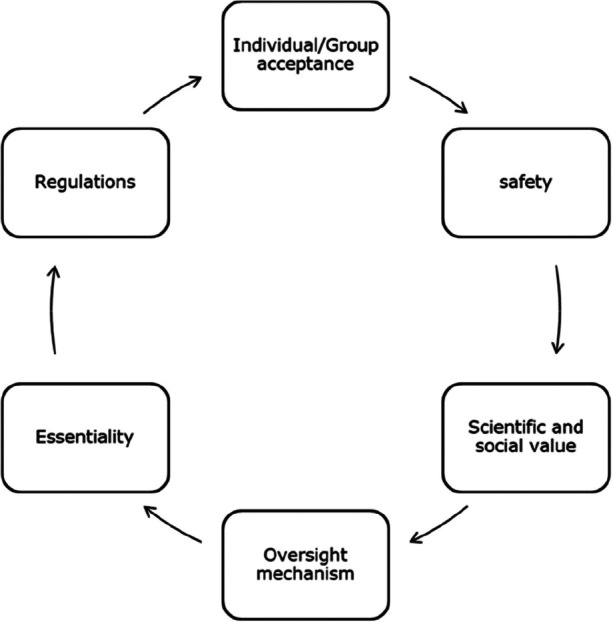
A model for consideration of partial disclosure as a research methodology

**Fig. 2:**
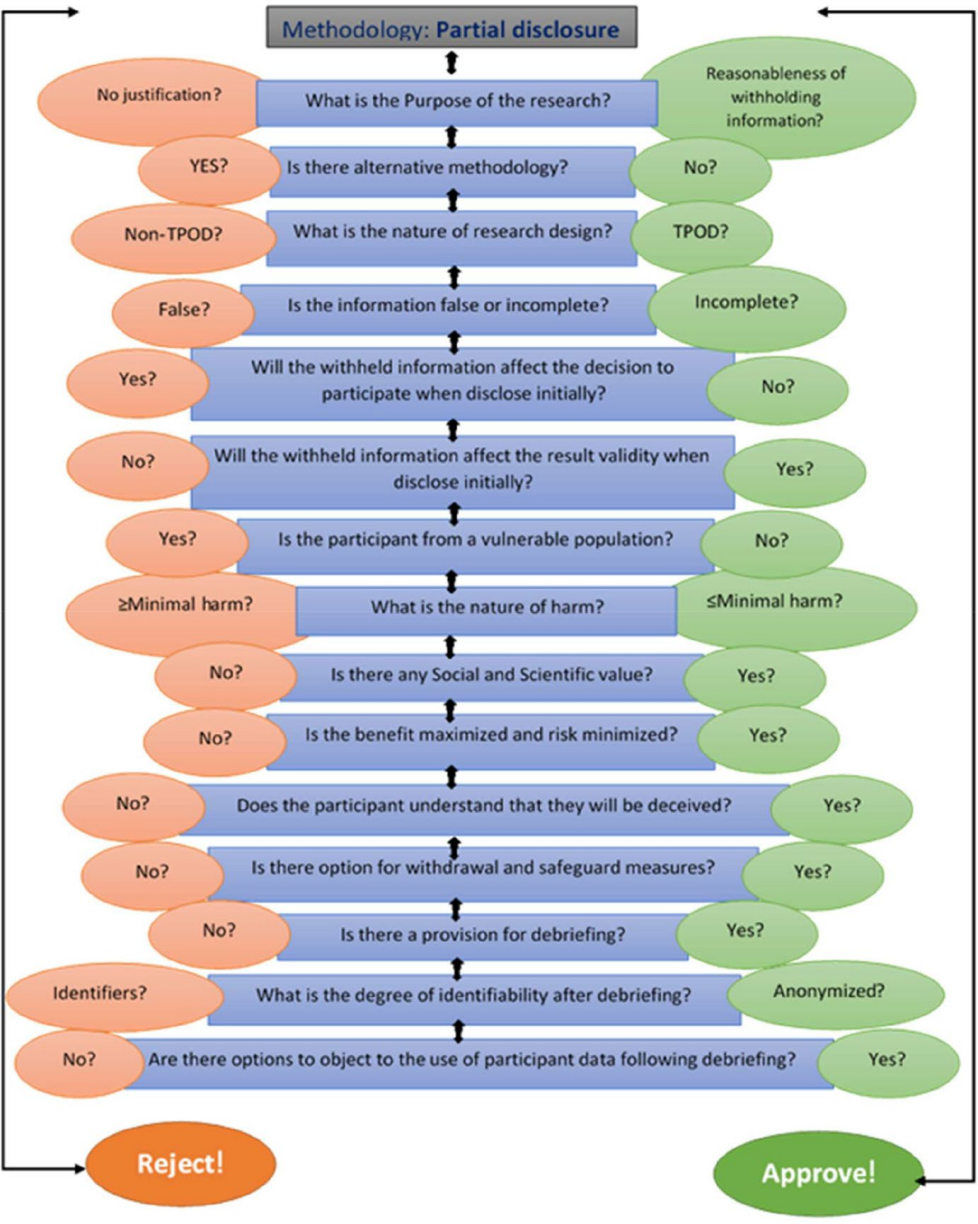
Iterative flowchart for assessment and approval of research involving partial disclosure. TPOD, therapeutic, preventive, observation, and diagnostic. Left-hand side > minimal harm (greater than minimal harm). Right-hand side < minimal harm (less than minimal harm)

Drawing from comprehensive reviews of literature, academic research, pertinent case studies on deception, and interviews with Ethics Committee members aligning with the research objectives were carried out. Additionally, during interviews, participants were queried about standard operating procedures (SOPs) pertaining to partial disclosure research and the potential components of such SOPs. Subsequently, utilising the collated and reviewed data, we crafted an SOP and a model, incorporating input from external members not directly involved in the study. Their insights were carefully considered in the refinement of the model and SOP ensuring robustness and adaptability. This proposed model (Fig. 1) demonstrates the practical and ethical considerations of partial disclosure in research without assuming one element has the upper hand; rather, it links each piece to get researchers closer to the moral principles underpinning ethical perspectives. Allowing for flexibility and adaptability, enabling adjustments in response to evolving insights, regulatory changes, or emerging best practises in research. The iterative flowchart for partial disclosure research and standard operating procedures (SOPs) presented in Fig. 2 encapsulates both practical and ethical considerations without favouring any single element, fostering a holistic approach towards moral principles governing ethical perspectives. This framework outlines a dynamic process that can be continually refined and enhanced over time, delineating steps for conducting partial disclosure research and formulating SOPs to ensure consistent and effective practises. Highlighting the importance of clear objectives in research, delineation of information to be partially disclosed, and the extent of disclosure.

### The Iterative Flowchart for Partial Disclosure Research and SOPs

The iterative flowchart for partial disclosure research and standard operating procedures (SOPs) involves steps that can be continuously refined and improved over time. The flowchart outlines conducting partial disclosure research and developing SOPs to ensure consistent and effective practises. The flowchart for partial disclosure research and SOP emphasises clear objectives and goals, the type of information to be partially disclosed, and the level of disclosure that will be made.

We comprehensively reviewed existing literature, academic research, and relevant case studies on deception to determine the key variables and factors pertinent to partial disclosure research. Also, conducted interviews with Ethics Committee members, participants were asked questions regarding SOP on partial disclosure research and the possible elements/components of such SOP. The SOP was written utilising the data collated from in-depth interviews; external members (uninvolved in the study) reviewed the analysed data for input, and comments were taken into consideration. The SOP and iterative flowchart are proposed for flexibility and adaptability, allowing for adjustments based on new insights, regulation changes, or emerging best practises.

### Proposed Standard Operating Procedure (SOP) for Partial Disclosure Research

In recognising that every protocol is distinct and requires individual consideration, assigning numerical values to safety could potentially oversimplify a nuanced evaluation process. Instead, this SOP prioritises adaptability, modification, interpretation, and reflection—crucial elements of ethical deliberation. Embracing flexibility and encouraging thoughtful deliberation, this definition of safety ensures that EC members can engage in a robust evaluation process that upholds ethical standards and promotes participant well-being. Hence, the SOP addresses concerns and provides descriptive and operational guidance for evaluating the safety of both participants and researchers and assigning social value. In the SOP, safety refers to the protection of participants, researchers, and other stakeholders from physical, psychological, and social harm or risk during the research process. This encompasses measures to minimise potential adverse effects and ensure the overall well-being and rights of all involved parties. The SOP acknowledges the dual safety concerns of both participants and researchers. While prioritising participant safety is paramount, EC members should also consider researchers’ ethical obligations and well-being, ensuring the integrity of the research process and the protection of professional reputation. The SOP outlines transparent criteria for assessing the social value of research, including its potential contributions to knowledge advancement, public health improvement, and societal welfare. EC members are guided to evaluate the significance of these potential benefits in a consistent and objective manner, facilitating ethical review processes.

#### Purpose

Standard operating procedure (SOP) provides guidelines for conducting partial disclosure research ethically and responsibly. Partial disclosure research involves intentionally or unintentionally withholding information from research participants under certain conditions with no intent of misleading.

#### Scope

This SOP applies to all EC members, researchers, staff, and personnel involved in planning, conducting, and reviewing partial disclosure research studies within the academic institution.

#### Ethical Considerations

Partial disclosure research must be conducted in accordance with ethical principles and guidelines to protect the rights and well-being of participants. The following ethical considerations should be adhered to:

- Informed consent: Prospective participants should be fully informed about the nature and purpose of the study, including the potential for partial disclosure. Partial disclosure should only be used if it is necessary for the research and cannot be reasonably achieved through other means.
- Debriefing: After the study, participants should be provided with a thorough debriefing explaining the true purpose of the research and the reasons for any partial disclosure employed. Any misconceptions should be addressed, and participants should have the opportunity to withdraw their data if desired.
- Minimisation of harm: The potential harm caused by partial disclosure should be minimised. Researchers should carefully evaluate the risks and benefits of using partial disclosure and take steps that ensure participants’ safety and protection.
- Confidentiality: Participants’ identities and personal information should be kept confidential, and any data collected should be stored securely and anonymised when possible.
- Independent Ethics Committee (IEC) or Scientific Review Board (SRB) Approval: Partial disclosure research as a methodology should be reviewed and approved by an appropriate Scientific Review Board or Independent Ethics Committee before initiation. Researchers must comply with this SOP set forth.

#### Study Design and Implementation

When designing and conducting partial disclosure research, the following considerations should be taken into account:

- Justification: Researchers must provide a clear and compelling rationale for employing partial disclosure in their study. The potential benefits of the research should outweigh the risks associated with partial disclosure.
- Design planning: Partial disclosure should be incorporated into the study design from the beginning, considering the specific research objectives, the nature and extent of disclosure required, and the measures to minimise harm.
- Partial disclosure methodology: Partial disclosure techniques should be carefully selected and justified based on the research objectives. Researchers should ensure that the partial disclosure is realistic, plausible, and does not cause severe distress or harm to participants.
- Monitoring and supervision: Studies involving partial disclosure should be closely monitored by EC members to ensure that the research is conducted appropriately and that any potential issues are addressed promptly.

#### Documentation and Reporting

Accurate documentation of the partial disclosure research process is essential for transparency and accountability. Researchers should maintain comprehensive records, including the following:

- Research protocols and procedures detailing the use of partial disclosure
- Informed consent forms and debriefing scripts clearly state the nature of partial disclosure
- Data collection instruments, analysis plans, and procedures for handling data
- Reports of any adverse events or participant concerns related to the partial disclosure

#### Training and Awareness

EC members reviewing the partial disclosure studies and the researchers involved in partial disclosure research should receive appropriate training and education regarding ethical considerations, potential risks, and best practises. Regular training sessions and discussions should be conducted to maintain awareness and ensure adherence to the SOP.

#### Review and Approval

This SOP should be reviewed and approved by the organisation’s research ethics committee or other relevant governing bodies. It should be periodically reviewed and updated as needed to reflect evolving ethical standards and regulatory requirements.

#### Compliance

All ECs and researchers and personnel involved in partial disclosure research are required to comply with this SOP. Non-compliance may result in disciplinary actions and could compromise the organisation.

Figure two shows the conditions under which using partial disclosure as a research methodology is acceptable. However, these processes are iterative, and the figure illustrates the anticipated flow of the decision-making process. The right (green colour code) shifts the decision in favour of approval, while the left (pink colour code) moves it in favour of rejecting the protocol.

## Study Limitation

The qualitative nature may restrict the generalizability of the findings. As qualitative research aims for in-depth understanding rather than statistical representation, the insights gained from the study may not be applicable to broader populations but can be understood in a particular context. Additionally, the small sample size of only eight participants may limit the diversity of perspectives and experiences captured in the study, potentially impacting the comprehensiveness of the findings. Furthermore, the use of only three selected ethics committees for data collection may introduce bias and limit the representation of varied institutional practises and perspectives on partial disclosure in research. Another limitation pertains to the qualitative nature of the SOP analysed. Qualitative methods may inherently allow for a more nuanced exploration of phenomena; however, they also introduce the possibility of subjective interpretation. These limitations should be considered when interpreting and applying the study’s findings to broader research ethics contexts.

## Conclusion

Partial disclosure presents several challenges for ethics committees, and incomplete information can hinder their ability to grasp the broader context, including the disclosed information’s motivations, intentions, and potential consequences. Without a comprehensive understanding, assessing the ethical implications becomes challenging. A lack of established guidelines or SOPs specifically addressing partial disclosure can create inconsistency in decision-making. Ethics committees rely on established protocols to guide their assessments and recommendations. However, no clear procedures for handling partial disclosure can lead to subjective judgements and varying approaches amongst committee members. This lack of uniformity may undermine the credibility and effectiveness of the committee’s decisions.

Partial disclosure is often ethically justified based on value, validity, and veracity considerations (3Vs). Partial disclosure is morally acceptable in preserving the value, upholding the validity, and maintaining the veracity or truthfulness of research findings. However, it is essential to consider the foundation of collective ethical positions or concerns associated with partial disclosure. Safety, essentiality, and beneficence are also other important ethical concerns that should be weighed against the reasons for partial disclosure. The decision to disclose partially should be based on careful ethical analysis, considering the specific context, potential consequences, and the overall impact on affected individuals or society. Ultimately, the ethical permissibility of partial disclosure should be evaluated on a case-by-case basis, taking into account the specific circumstances, the ethical principles involved, anticipated benefits, and anticipated harms associated with the decision.

## Data Availability

The datasets generated from the interviews conducted during the research, as well as the coded datasets from the analysis, will be made available in anonymized form upon request via the corresponding email.
